# Goldilocks Meets Santa Rosalia: An Ephemeral Speciation Model Explains Patterns of Diversification Across Time Scales

**DOI:** 10.1007/s11692-012-9171-x

**Published:** 2012-03-14

**Authors:** Erica Bree Rosenblum, Brice A. J. Sarver, Joseph W. Brown, Simone Des Roches, Kayla M. Hardwick, Tyler D. Hether, Jonathan M. Eastman, Matthew W. Pennell, Luke J. Harmon

**Affiliations:** 1 Department of Biological Sciences, University of Idaho, Moscow, ID 83844 USA; 2 Department of Environmental Science, Policy, and Management, University of California, Berkeley, CA 94720-3114 USA; 3 BEACON Center for the Study of Evolution in Action, East Lansing, MI USA

**Keywords:** Adaptation, Incipient speciation, Geographic isolation

## Abstract

Understanding the rate at which new species form is a key question in studying the evolution of life on earth. Here we review our current understanding of speciation rates, focusing on studies based on the fossil record, phylogenies, and mathematical models. We find that speciation rates estimated from these different studies can be dramatically different: some studies find that new species form quickly and often, while others find that new species form much less frequently. We suggest that instead of being contradictory, differences in speciation rates across different scales can be reconciled by a common model. Under the “ephemeral speciation model”, speciation is very common and very rapid but the new species produced almost never persist. Evolutionary studies should therefore focus on not only the formation but also the persistence of new species.

How often do new species form? Studies of plant and animal speciation rates have focused on different species, over different time scales, using different methods (e.g., Simpson 1944; Givnish 2000; Coyne and Orr 2004). Here we review our current understanding of speciation rates, focusing on reconciling what we know from studies of speciation based on the fossil record, phylogenies, and mathematical models. We find that speciation rates estimated from these different studies can be contradictory, with some rates clearly much faster than others. Given that some rates seem “too slow” and some seem “too fast”, is there a single framework that predicts rates that are “just right”? To reconcile results from different approaches, we highlight an “ephemeral speciation model”, under which new species form frequently but rarely persist.

## Background

The most common way to estimate rates of speciation is to use data from the fossil record. *Paleontological studies* estimate how many new species formed over a given time interval (the per lineage speciation rate; e.g., Stanley 1979; Van Valen 1985; Jablonski 1986; Hulbert 1993; Sepkoski 1993). Many paleontological estimates of speciation rates have been calculated from groups with reasonably complete fossil records like marine invertebrates (e.g., Raup and Sepkoski 1982; Peters 2005). For groups with incomplete fossil records, sophisticated statistical analyses can correct for incomplete sampling (e.g., Alroy et al. 2008; Foote 2000; Ezard et al. 2011). Paleontological approaches for inferring speciation rates use direct information about species that lived in the past, whereas other methods (discussed below) must infer the past dynamics indirectly. The primary limitation of paleontological studies is the uncertainty that arises from gaps in the fossil record (e.g., limited specimen material, uneven sampling effort, and/or taphonomical biases; Raup 1979; Sepkoski 1998; Alroy et al. 2008). Furthermore, paleontological studies generally rely on higher taxa (Valentine 2004), and how these taxonomic groups are defined can have a profound influence on inferred speciation rates (Ezard et al. in press).

Paleontological studies suggest that speciation rates vary widely both through time and across lineages. Most paleontological estimates of per-lineage speciation rates range from 0.01 to 10 speciation events per lineage per million years (Sepkoski 1998); Sepkoski (1998) suggests a “canonical” estimate of 0.3 speciation events per lineage per million years. Even though this rate varies tremendously both across taxa and through time (Sepkoski 1998), it can serve as a rough but useful “benchmark” for comparisons across datasets.

Speciation rates can also be inferred from *phylogenetic studies* of extant species. This approach requires phylogenies whose branch lengths have been scaled to time. The simplest way to calculate speciation rates from these trees is to compare species richness to clade age, which provides a minimum bound on the rate of speciation, assumed to be constant (Magallon and Sanderson 2001; but see Rabosky 2009, 2010 who warns against this approach). Another approach is to fit models (i.e., birth–death models) to phylogenetic branch lengths and estimate rates of speciation and potentially extinction (reviewed in Nee 2006). Using phylogenies of extant species takes advantage of the wealth of data from the tree of life (Hedges and Kumar 2009). However, phylogenetic trees are estimated with error, do not include direct information about extinct species, and suffer from a number of biases related to sampling, all of which can affect speciation rate estimates (e.g., Revell et al. 2005; Phillimore and Price 2008; Rabosky 2010; Cusimano and Renner 2010; Brock et al. 2011).

Despite these potential caveats, most phylogenetic studies of speciation rates recover estimates that are of the same order of magnitude as the fossil record. One meta-analysis of speciation rates estimated from phylogenies found rates that ranged from 0.01 to 10 speciation events per lineage per million years under a pure birth model (McPeek and Brown 2007). An approach that estimated both speciation and extinction rates simultaneously recovered speciation rates of the same order of magnitude (e.g., Alfaro et al. 2009).

In contrast to the studies discussed above, many studies of young evolutionary radiations estimate very high rates of speciation. These estimates often come from adaptive radiations in insular habitats (e.g., islands and lakes; see Losos and Ricklefs, 2010, and chapters therein). For example, one of the best-known examples of rapid diversification is the cichlid fishes in lakes of the African Rift Valley (Seehausen 2006). Estimated speciation rates for cichlid species flocks are quite high. An extreme example occurs in Lake Victoria, where speciation rates might be as high as 400 speciation events per lineage per million years (i.e., the formation of ~450 species in ~15,000 years, Johnson et al. 1996; Genner et al. 2004; but see Day et al. 2008). Other studies suggest that similarly high speciation rates may occur in other systems [e.g., Hawaiian drosophila (Coyne and Orr 2004), silverswords (Baldwin and Sanderson 1998)]. In fact, there are several well-known adaptive radiations that have occurred so quickly, it is extremely difficult to infer the true phylogenetic relationships among species [e.g., Galapagos finches (Sato et al. 1999; Petren et al. 1999; Burns et al. 2002; Grant and Grant 2007), three-spined sticklebacks (reviewed in Schluter 2000)].

Concordant with studies of young evolutionary radiations, *mathematical models of speciation* also suggest that speciation rates can be quite high. Mathematical models of speciation take a number of forms, including models of sympatric speciation (e.g., Maynard-Smith 1966; Felsenstein 1981; Dieckmann and Doebeli 1999; Doebeli and Dieckmann 2003), models of divergence with gene flow (e.g., Wu 2001; Hausdorf 2011), models of speciation in allopatry (e.g., Gavrilets 2003), and metapopulation models of adaptive radiations (e.g., Gavrilets and Vose 2005). Modeling approaches allow for a mechanistic understanding of how specific parameters influence the process of speciation, but are obviously limited by their simplifying assumptions. For example, models of speciation make specific assumptions about population structure, genetic architecture, and the strength and type of selection. Additionally, mathematical models must necessarily assume a relatively simplistic species concept. Model assumptions and parameter values can have dramatic impacts on the dynamics of speciation models (reviewed in Gavrilets 2004).

Mathematical models of speciation almost never focus on speciation rate *per se*, and instead generally ask whether or not speciation occurs and, if so, how long it takes from start to finish (i.e., the speciation “transition time”, Coyne and Orr 2004). Transition times estimated from mathematical models are typically very fast (e.g., Doebeli and Dieckmann 2003, but see Orr and Turelli 2001). However, transition times are not directly related to speciation rates, which describe the time from one speciation event to the next (i.e., the speciation “waiting time”). One modeling study that focused explicitly on speciation rates found that when speciation occurred, the waiting time for speciation varied between 5,000 to more than 200,000 generations depending on model parameters (Gavrilets 2000). For organisms with a generation time of 1 year, this corresponds to speciation rates of 2–200 speciation events per lineage per million years. These rates are comparable to the highest speciation rates observed empirically in the young evolutionary radiations discussed above, and likely represent an upper bound for speciation rates. It is difficult to define a lower bound for speciation rates from mathematical model because these models typically do not focus on parameter values where speciation never happens.

## Ephemeral Speciation Model

Speciation rates estimated from studies at different time scales suggest a contradiction. Mathematical models of speciation and studies of young evolutionary radiations find that new species can form quickly and often. However, phylogenetic studies over longer time scales and paleontological studies find that new species usually form more slowly. What explains this apparent discrepancy in speciation rates across different types of studies?

Here we call attention to an explanation that may help unify what we know about speciation rates from paleontological, phylogenetic, and mathematical studies: the “ephemeral speciation model”. It is possible that speciation is very common and very rapid, but that the new species produced almost never persist. Therefore we suggest that some approaches (e.g., studies of speciation in action and mathematical models) actually focus on the formation of ephemeral species while others (e.g., phylogenetic studies over longer time scales and paleontological studies) focus on the persistence of these ephemeral species. Instead of being a contradiction, these differences in speciation rates reflect two aspects of the same underlying model: the formation and the persistence of ephemeral species.

The concept of fragile new species has deep historical roots. The idea that many more incipient species form than persist traces back to Mayr (1963). Stanley (1978, 1985) also discussed this phenomenon, referring to these failed incipient species as “aborted species” (Almon 1992). Levin (2000, 2005) proposed a related idea for plants where many incipient species form and have differential survival (i.e., “isolate selection”). Other authors have discussed characteristics necessary for “successful speciation” (e.g., geographic range expansion and ecological niche differentiation (Price 2008; Rundell and Price 2009). Hubbell’s neutral theory of ecology also exhibits high turnover of young “incipient” species (Hubbell 2001; Rosindell et al. 2010; Etienne and Rosindell 2011). Our suggested name—“the ephemeral speciation model”—takes inspiration from Futuyma’s ephemeral diversification model (which focuses primarily on trait change: Futuyma 1979, 2010).

**Fig. 1:**
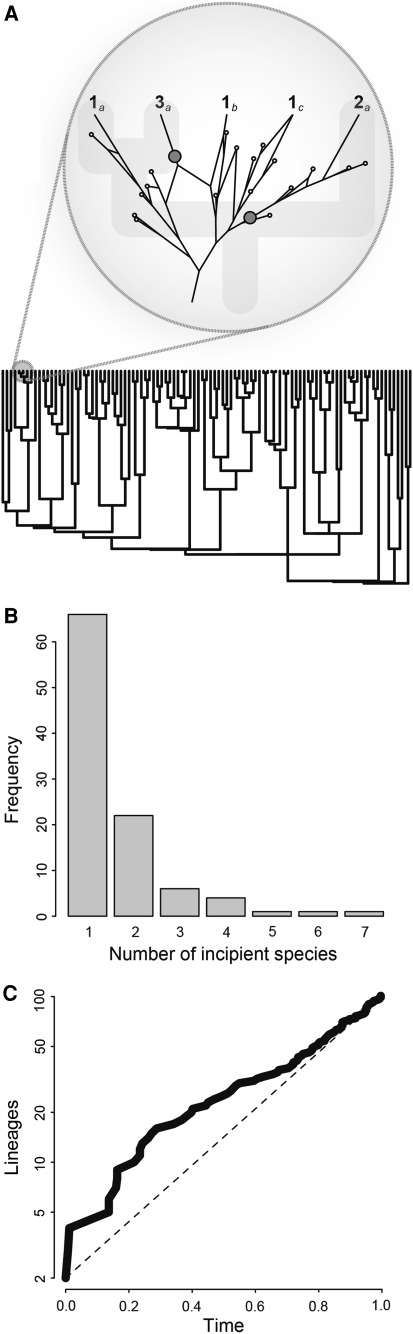
Simulation of a hierarchical model of ephemeral speciation. The model has three parameters: the incipient speciation rate, the incipient extinction rate, and the rate of formation of “full species”. Simulation results are consistent with the core qualitative predictions presented in the text. (**a**) A phylogenetic tree showing that species are composed of many incipient forms, most of which go extinct or are reabsorbed via hybridization (*inset*). (**b**) A frequency distribution showing the uneven distribution of incipient forms within species. The x-axis shows the number of incipient species contained in each “full species”. (**c**) A lineage through time plot showing an “early burst” of speciation due to the preferential survival of clades that form many new species by chance early in their history

Under an ephemeral speciation model, new incipient species are constantly forming at a high rate (Fig. 1). Recent research on mechanisms of speciation suggest that speciation can occur via a plurality of interacting mechanisms (e.g., geography, selection, genomic rearrangements; see Coyne and Orr 2004; Gavrilets 2004). Thus the conditions for some mode of speciation may often be met in natural populations. Although speciation occurs frequently in the ephemeral speciation model, persistence of incipient species is rare. The lack of persistence could be due to extinction or “reabsorption” via hybridization of the incipient species (Seehausen et al. 1997; Seehausen 2006; Taylor et al. 2006; Richmond and Jockush 2007; Behm et al. 2010). For example suppose that in some clades speciation typically happens in small allopatric populations at the edge of a species range (Mayr 1963). These new species will be very vulnerable to extinction and to changes in the conditions that maintain reproductive isolation (Mayr 1963). New species are also likely to be fragile under other non-allopatric modes of speciation as well [e.g., new polyploid species have very small population sizes (Holloway et al. 2006) and new ecological species require continued divergent selection until other forms of reproductive isolation evolve (Nosil and Sandoval 2008)]. Therefore failed speciation is common because speciation takes time to complete and because conditions change over time.

The ephemeral speciation model is consistent with two key empirical observations. First, what taxonomists recognize as species are often comprised of many incipient forms. Species typically show extensive genetic and phenotypic variation, and this variation is usually hierarchically structured (Avise 2000; Bickford et al. 2007; Mallet 2008). These incipient forms are recognized taxonomically by a variety of names (e.g., geographic races, subspecies, incipient species; Simpson 1944; Mayr 1982). Here we follow the general lineage concept of species, which encompasses many different species concepts as points along a continuum and which is consistent with the idea that species themselves can be a collection of distinct lineages (De Queiroz 2005). Second, new species can be unstable. When incipient species form, they can go extinct or cause their parental forms to go extinct (e.g., Hegde et al. 2006). Additionally, incipient species can collapse and be reabsorbed by their parental form (Mallet 2008). One recent example is the collapse of a stickleback species pair in Enos Lake (Taylor et al. 2006; Behm et al. 2010), but similar collapses of incipient species have been observed in cichlids (Seehausen 2006); trout (Bettles et al. 2005), Galapagos finches (Grant and Grant 1993, 2008); skinks (Richmond and Jockusch 2007) and house spiders (Croucher et al. 2007).

One way to model ephemeral speciation over long time scales is to use a simple high turnover birth–death model with high speciation and high extinction rates (e.g., Alfaro et al. 2009). These high turnover models do a good job of predicting species diversity in clades of intermediate age [e.g., jawed vertebrates (Alfaro et al. 2009) and ray-finned fishes (Santini et al. 2009)]. But high turnover birth–death models are inconsistent with empirical data in three ways. First, high turnover models predict that species deep in the tree should accumulate exponentially through time (i.e., linear lineage through time plot, Nee et al. 1992), but this pattern is not commonly seen in phylogenetic trees (Rüber et al. 2005; Phillimore and Price 2008). Second, high turnover models suggest that the persistence of incipient species should be random, but empirical work on persistence of populations across a species range suggests that population survival is not typically random with respect to both biotic and abiotic factors (e.g., Levin 2000, 2005; Owens et al. 2000; Jones et al. 2003; Cardillo et al. 2008). Third, in high turnover models, species fail to persist only because they go extinct, but incipient forms can fail to persist because they are reabsorbed. Therefore, more elaborate models of ephemeral speciation are needed.

We suggest that ephemeral speciation models should be hierarchical (Gould 1980) where incipient forms are constantly forming and dissolving inside larger entities (i.e., species, Fig. 1a). There are several existing models that consider a hierarchical process where “incipient” forms arise and go extinct at a higher rate than “full” species (Cadena et al. 2005; Pons et al. 2006; Phillimore et al. 2007; Pigot et al. 2010). The most general example is Phillimore et al. (2007) who find that the rate of bird “subspeciation” is between 30 and 40 times higher than the rate of speciation (see also Martin and Tewksbury 2008).

There are several important consequences of hierarchical models of ephemeral speciation (Fig. 1). First, species go extinct only when all incipient forms are lost. Therefore the extinction rate is no longer a property of a species but depends on the number of incipient forms within the species and their extinction rate. Second, like other hierarchical models (e.g., Wakeley 2008; Hubbell 2001), the ephemeral speciation model predicts an uneven distribution of incipient form within species (Fig. 1b): a few species will contain many incipient forms while most species will contain few (consistent with the observation that rare species are common; Lim et al. 2011). Third, this uneven distribution will lead to differences in effective speciation and extinction rates across species. Species with many incipient forms will have high speciation and low extinction rates compared to species with few incipient forms (see also Kisel and Barraclough 2010). The unevenness of speciation rates across taxa is consistent with the fact that phylogenetic trees tend to be more unbalanced than birth–death models predict (Mooers and Heard 1997). Finally, under the ephemeral speciation model it is very likely for entire clades of newly formed species to go extinct. The clades that survive to the present day are disproportionately likely to have undergone a burst of speciation early in their history (Phillimore and Price 2008), which could be an explanation for observed slowdowns in lineage accumulation through time (see also Pigot et al. 2010, Fig. 1c).

Finally, the conceptual link between the ephemeral speciation model and the ephemeral divergence model (Futuyma 1979; Futuyma 2010) reflects a common pattern for rates of trait evolution and rates of speciation. New incipient forms are constantly arising within species. Similarly, traits are constantly changing in response to local selection pressures and/or drift. In both cases these changes rarely persist over long time scales. It is important to note that we are not arguing for a model of punctuated equilibrium; trait change may or may not be associated with the formation of incipient species (Bokma 2008). The important point is that most of the change that occurs over short time periods does not last. Therefore a fundamental shift suggested by both ephemeral models is that evolutionary studies of diversity patterns should focus on not only the formation but also the persistence of new traits and species [e.g., why do some some species persist and others perish quickly? (Levin 2000, 2005; Weir and Schluter 2007; Martin and Tewksbury 2008; Stanley 2008)].

Although some discussion of the fragile nature of species has occurred in the evolutionary biology literature over the last 50 years, we suggest that the idea of ephemeral speciation has not been deeply incorporated in the way scientists think about speciation. Following Hutchinson (1959), evolutionary biologists have often referred to Santa Rosalia when asking why are there so many or so few species on Earth (e.g., Felsenstein 1981). We suggest that the ephemeral speciation model provides a resolution to the Goldilocks paradox of species diversity: the balance between rapid species formation and rare persistence can explain why the number of species on Earth is “just right”.
